# Exploring the correlates and nature of subjective anomalous interactions with objects (psychometry): a mixed methods survey

**DOI:** 10.3389/fpsyg.2024.1365144

**Published:** 2024-09-02

**Authors:** Christine A. Simmonds-Moore

**Affiliations:** Department of Anthropology, Psychology and Sociology, University of West Georgia, Carrollton, GA, United States

**Keywords:** psychometry, anomalous experiences, synesthesia, mixed methods research, autonomous sensory meridian response (ASMR)

## Abstract

**Introduction:**

Psychometry refers to the experience of receiving information about a person or thing by contact with a given object. There is little research to date on the psychological correlates of psychometry and no systematic qualitative research on the nature of the experience itself.

**Method:**

A convergent mixed methods online survey sought to explore how synesthesia and autonomous sensory meridian response (ASMR) correlate with a range of anomalous experiences, including psychometry, among members of the public. Those who reported that they had experienced psychometry were invited to describe their experiences in an open ended section.

**Results:**

Results indicate that those who experience psychometry scored higher on a measure of ASMR than those who did not. Those who experience synesthesia also scored significantly higher on a measure of ASMR than those who did not. However, synesthesia was not significantly associated with psychometry. Both ASMR and synesthesia were associated with tendencies to report anomalous experiences (with and without a paranormal attribution). A thematic analysis found five themes including: a flash of imagery; lived feelings and intense emotions; noesis and perspective taking/empathy. Subjective psychometry experiences seem to reflect emotional information that is experienced as different to one’s normal experiences and felt to be from the perspective of another person.

**Discussion:**

Results are discussed and quantitative and qualitative findings are integrated.

## Introduction

Psychometry has been defined as “the practice of using an object from a person, such as a piece of jewelry or clothing, to obtain information about the person” (Roll, 2003, p. 201). It has also been referred to as “a type of clairvoyance facilitated by the handling of an object” (Barrington, 2016, p. 1). The term originates from the Greek word psyche (soul) and metron (measure) (Buchanan, 1885). Psychometry was systematically considered in the early 19th Century by Buchanan (1885) and Denton and Denton (1871) followed by research conducted by prominent members of the Society for Psychical Research and the American Society for Psychical Research, including Edmund Gurney and William James. Early studies by psychical researchers tended to explore the claims of self-professed mediums (Hettinger, 1941, 1948; Dale, 1944; e.g., Pagenstecher, 1920). Other systematic studies examined the psychometric abilities of special claimants by Osty, Poniatowski and Richet (cited in Barrington, 2016). Research exploring psychometry and the use of token objects in psi studies^1^ continued into the 1960s, 1970s and 1980s (Roll, 1966; LeShan, 1967; Tart and Smith, 1968; Smythies, 1987; Saklani, 1988). The topic has been relatively neglected until recently and academic attention has been redirected toward the study of psychometry (Parra and Argibay, 2007, 2008, 2009; Baker et al., 2017). To date, however, there is little research on individual difference correlates of subjective experiences with psychometry or how subjective experiences of psychometry relate to other anomalous experiences. There also a need for systematic qualitative explorations of these experiences. This project seeks to better understand the correlates and nature of self proclaimed experiences with psychometry and other subjective paranormal experiences.

### Psychometry and related phenomena

Anomalous experiences reflect a range of experiences that fall outside of the typical or usual ways in which consciousness, the sense of self and reality are experienced (Cardeña et al., 2017) and include experiences labeled as “paranormal” and “parapsychological”. There are three main categories of subjective parapsychological experience, which include anomalous information access (extrasensory perception), anomalous mind-matter interactions^2^ and experiences reflecting experiences suggestive of a separation of human consciousness from the physical body (the “survival hypothesis”) (Vernon, 2021). Psychometry experiences consist of physically interacting with objects and experiencing psychic impressions pertaining to their previous owner. It is often practiced by self-proclaimed mediums^3^ in relation to accessing information about the deceased but is also practiced in the context of accessing other types of information (including information about the living). Psychometry experiences could therefore fit under the category of extrasensory perception experiences, given that some have argued that the practice of psychometry reflects a way to focus one’s psychic attention. Others argue that information (about the living or deceased) is connected to a physical object (*cf.*, Rogo, 1974), which may fit the category of an anomalous mind-matter interaction experience. Others have argued that psychometry provides evidence for the survival hypothesis, suggesting the persistence of human consciousness beyond bodily death (Anderson, 1984).

Psychometry experiences relate to several other subjective experiences and paranormal claims akin to a family tree of related phenomena. These include experiences in which physical objects or tokens are provided to a person who tries to psychically access information at a distance (or Remote Viewing, e.g., Tart and Smith, 1968). Psychometry experiences also relate to haunting experiences whereby a set of experiential phenomena occur in a particular location and are attributed to presence of a ghost (Houran et al., 2019). Likewise, poltergeist experiences are characterized by anomalous movements of physical objects (Houran et al., 2019). Sometimes these anomalous experiences appear to focus on some objects more than others (Roll and Joines, 2013). Anomalous heart transplant experiences are also relevant to experiences of psychometry. Here, claimants report personality changes following a heart transplant which can sometimes seem to align with the personality and memories of the donor, suggesting a biological form of psychometry experience (See Pearsall et al., 2002; Liester, 2020). Given its association to other anomalous experiences and claims regarding paranormal phenomena, it will be interesting to understand how psychometry relates to other forms of anomalous experiences, how psychometry experiences correlate with some individual difference measures and what these experiences are like. We therefore take a mixed methods research approach and include quantitative and qualitative aspects in our research design.

### The psychology of psychometry

To date, the majority of research that has investigated psychometry has tended to focus on testing the claims of psychic claimants and mediums (Denton and Denton, 1871; Buchanan, 1885; Pagenstecher, 1920; Hettinger, 1941, 1948; Dale, 1944; Roll, 1966; LeShan, 1967; Smythies, 1987; Strang, 2020). Recent research has explored performance at a psychometry task among members of the general population (Parra and Argibay, 2007, 2008, 2009; Baker et al., 2017). However, there has been little systematic research exploring the psychological correlates and phenomenology of subjective experiences of psychometry.

Early theorists noted that passiveness of mind seemed to be an important trait of the psychometrer (see Strang, 2020). Strang (2020) has also noted that during 19th century Spiritualism, it was assumed that psychometry worked better among those who are more sensitive and receptive to information. This was assumed to be a particular facet of females, who often took the role as the psychometrist. In a discussion of psychometry in the Halls Journal of Health (Science of Psychometry: Phenomenal Perceptions, 1889) it was noted that people who are prone to these experiences are more susceptible to various subtle aspects of the world, including changes in the atmosphere (weather). This suggests a certain level of psychological sensitivity among psychometry experiencers. Sensitivity has been consistently associated with other forms of subjective paranormal experiences, including apparitions (e.g., Jawer, 2006). This extends to a range of other anomalous experiences, for example, in correlation with the transliminality variable (Lange et al., 2019). The current definition for transliminality is a “hypersensitivity to psychological material originating in (a) the unconscious, and/or (b), the external environment” that is understood to reflect heightened neuroplasticity, or the tendency to make physiological and psychological connections for information that may derive from a variety of sources. At the heart of transliminality is a greater sensitivity to psychological material from external or internal sources. Psychological materials are also more likely to be fused via syncretic processes, including eidetic imagery, physiognomic perception and synesthesia. Psychometry may also relate to the related construct of sensory processing sensitivity, which has previously been associated with subjective paranormal experiences (Williams and Blagrove, 2022). In support of an association between sensitivity and psychometry experiences, previous research has connected transliminality to other object-related anomalous experiences including poltergeist phenomena (Ventola et al., 2019) and hauntings (Laythe et al., 2021).

Other research suggests a role for an altered state of consciousness when people practice psychometry. For example, Pagenstecher’s (1920) studies focused on evaluating the claims of a Mexican medium who seemed to be able to gain access to information by entering a hypnotic-like trance state and touching the object with her fingertips. It is reported that Senora Z experienced visual impressions, auditory experiences and emotional reactions in response to touching the objects and that the information that she received was heralded to be accurate. Some of the descriptions provided by the author suggest that Senora Z experiences the information as if she is present and from a first person perspective. Another study explored psychometric abilities in a person who was under the influence of mescaline, and found some support for the psychometry hypothesis (Smythies, 1987). This aligns with other research that has found an association between alterations in consciousness and paranormal experiences (*cf.* Cardeña, 2020). Another study found that mediums who experienced psychometry (among other psychic phenomena) demonstrated tendencies to be “self-sufficient,” “undisciplined,” and “affected by feelings” (Hearne, 1989). This suggests that a person who reports subjective experiences of psychometry is more emotionally labile or influenced by the emotions of others, which may indicate some forms of empathy or heightened responsiveness to emotions.

### Individual difference correlates of psychometry experiences

Little research has explored individual difference correlates of psychometry experiences. Our study seeks to explore the psychology of these experiences in a larger sample from the general population. We particularly focus on autonomous sensory meridian response (ASMR) and its rarer cousin, synesthesia, in relation to subjective experiences of psychometry. In the next section, we discuss our rationale for exploring these variables in relation to subjective psychometry experiences and to a wider measure of anomalous experiences.

#### ASMR

ASMR reflects “the experience of tingling sensations in the crown of the head, in response to a range of audio-visual triggers such as whispering, tapping, and hand movements” (Poerio et al., 2018, p. 1). ASMR videos are commonly used among younger individuals with the intention of eliciting a state of relaxation and promoting sleep (*cf.* Sakurai et al., 2021). In general, ASMR appears to be associated with subjective and objectively measured positive mood states, relaxation and appears to be similar to states of absorption including the flow state (Engelbregt et al., 2022). Neural imaging studies support a genuinely physiological influence of ASMR in terms of changes in brain activity (increases in alpha) indicative of a meditative or flow like state that also activates motor and sensory areas (gamma wave activity) (Fredborg et al., 2021). Overall, tactile and interpersonal triggers tend to be the strongest at eliciting an ASMR response (Poerio et al., 2023). A recent study found that the prevalence rate for ASMR responders is 20% in the population (Poerio et al., 2022b). There is also evidence that ASMR seems to be part of a family-tree of phenomenologically related experiences, including frisson (an emotional and embodied response to music, which also includes a chill or tingling sensations that may move up or down the body, potentially spreading to the chest, abdomen and legs) and misophonia (a given external stimulus, often eating or chewing is associated with negative emotions). Research indicates that those who report ASMR are more likely to report misophonia (Janik McErlean and Banissy, 2018) and experience frisson (del Campo and Kehle, 2016; Roberts et al., 2020). ASMR has yet to be explored in relation to subjective paranormal experiences, but seems to be promising as an *anomaly-prone* variable, given its association with several traits that are associated with increased connectivity and sensitivity and tendencies to report anomalous experiences including openness to experience (Fredborg et al., 2017; Janik McErlean and Banissy, 2017), increased scoring on transliminality (Roberts et al., 2021), sensory processing sensitivity (Poerio et al., 2022a), interoceptive sensitivity (body consciousness) (Roberts et al., 2021; Poerio et al., 2022a), higher scores on empathic concern (Janik McErlean and Banissy, 2017) and higher scores on a measure of positive schizotypy (unusual experiences; Roberts et al., 2021). ASMR has also been empirically demonstrated to be associated with increased sensory sensitivity (pain thresholds; Janik McErlean et al., 2022). Other research has found that ASMR is associated with greater connectivity between different areas of the brain (Smith et al., 2017). In addition, ASMR experiences seem to be associated with “a reduced ability to inhibit sensory-emotional experiences that are suppressed in most individuals” (Smith et al., 2017) indicative of more fluid psychological boundaries and characteristic of transliminality (described earlier). This might result in more availability of emotional experiences that might then be given a paranormal attribution.

ASMR may play a role in the etiology of psychometry experiences due to the interpersonal context of many ASMR triggers (Barratt et al., 2017). Its association with trait empathy might suggest that psychometry experiences could potentially arise in the context of imagined others when one is handling an object that was once owned, or worn by another person. This is a viable explanation, given that empathy can occur in the context of directly observing or imagining the experiences of social others (Lamm et al., 2016).

#### Synesthesia

Synesthesia occurs when there is an additional response (or concurrent) to an inducing stimulus (the inducer) and has previously been linked to enhanced reporting of a range of anomalous and paranormal experiences (Simmonds-Moore et al., 2019). For example, one common form of synesthesia is grapheme–color synesthesia in which a person experiences a specific color in response to a specific stimulus, e.g., the color purple in response to the letter S. Congenital or strong synesthesia is reported by approximately 5% of the population and reflects experiences that are automatic and consistent and which have been experienced since childhood (Simner and Hubbard, 2013). There are phenomenological differences in how people experience the concurrent. For some, the concurrent is projected out into the external world among “projectors”, while for others it is experienced internally among “associators” (Alvaraz and Robertson, 2013), while others experience the concurrent in more complex ways (Eagleman, 2012). Cytowic (1995) observed that synaesthetes seem to experience a lot of subjective paranormal experiences, including experiences of déjà vu, clairvoyance, precognition (in dreams), sense of presence, empathic healing and psychokinesis. Others have claimed that synaesthesia may actually underpin anomalous experiences as a kind of “building block” (e.g., Irwin, 1985; Alvarado, 1994; Williams, 1997; Marwaha and May, 2015). Research supports an association between synesthetic tendencies and subjective parapsychological experiences and with general tendencies toward reporting anomalous experiences (Simmonds-Moore et al., 2019). Synesthesia may facilitate a range of anomalous experiences by providing a tangible representation for a range of stimuli that usually fall outside of conscious awareness, including unconscious influences and environmental stimuli such as geomagnetism (*cf.* Simmonds-Moore, 2022).

#### ASMR is synesthesia-like

ASMR tendencies are also associated with enhanced prevalence rates of synesthesia (Barratt and Davis, 2015) and may reflect a form of synesthesia, or synesthetic tendencies (Janik McErlean and Banissy, 2017; Poerio et al., 2022b). Research has shown that synesthesia seems to be associated with a greater likelihood of reporting ASMR experiences, and ASMR responders are four times more likely to also experience synesthesia (Poerio et al., 2022b). It is noted that this shared variance may be due to many factors, including a shared neural and cognitive architecture, an enhanced tendency to report anomalous experiences or that ASMR is a form of synesthesia in its own right. In this study, we explored synesthesia and ASMR tendencies in the context of subjective experiences with psychometry and general measures of tendencies to report anomalous experiences.

### Phenomenology of psychometry experiences

To complement the quantitative exploration of the correlates of psychometry experiences, a qualitative analysis was included in our study design to explore what these experiences are like in a convergent mixed methods design. There has been a recent movement toward adopting qualitative research approaches for subjective paranormal experiences (e.g., Kruth, 2015) but few studies have adopted a mixed methods approach. Baker et al. (2017) used a mixed methods design in their own investigation of psychometry, which enabled greater insights into the reasons for statistical differences between a group of psychic claimants and non claimants on a psychometry task. It is of academic interest to explore how people describe their experiences with psychometry in order to further explore the reasons why people report subjective experiences regarding anomalous interactions with objects.

### Hypotheses and research questions

- ASMR scores will be higher for those who report psychometry
- Synesthetes will be more likely to report psychometry experiences compared to non-synesthetes
- ASMR scores will correlate with tendencies to report anomalous experiences
- ASMR scores will be higher among those who answer a question about synesthesia
- A qualitative question asked what are psychometry experiences like?

## Methods

### Design

This study was conducted using a convergent mixed methods design such that we could explore correlations and difference tests regarding ASMR and anomalous experiences, including psychometry and synesthesia and anomalous experiences including psychometry and unpack further details about the nature of psychometry experiences in a qualitative component. The quantitative component included measures pertaining to synesthesia, ASMR, anomalous experiences and psychometry while the qualitative component consisted of an open ended question that invited participants to share the details of any subjective experiences with psychometry. Planned analysis for the quantitative component were difference tests and correlations. The planned analysis for the qualitative section was thematic analysis. We planned to integrate the findings from the quantitative and qualitative aspects of the project in the discussion section of this paper.

### Participants

Following data cleaning,^4^ 164 participants completed the survey of which 40 were male and 112 were female. The most frequent age group was the 18–24 category, followed by 25–34, then 35–44. 45–54, 55–64 and finally an over 65 group. One hundred and thirty-eight participants were right handed, 16 were left handed and 10 were ambidextrous. The sample included 128 White, 16 Black, 5 Asian and 15 other ethnic groups.

### Materials

Several measures were uploaded into Qualtrics. The study URL may be viewed here https://westga.co1.qualtrics.com/jfe/form/SV_7OFgowuJSsJayLI. The different components of the survey are described below.

#### Consent statement

A consent document was uploaded into qualtrics, including the IRB approval number for the study. The consent statement included adherence to various ethical considerations including privacy and confidentiality, the right to withdraw from the study at any time, etc. Participants were asked to affirm their consent to participate by clicking yes. If they clicked no, they were redirected out of the survey.

#### Demographics

Participants were asked about gender identity, handedness and their age (assessed in terms of age categories). If participants were under the age of 18, they were redirected out of the survey.

#### Synesthesia question

Synesthesia was assessed by inviting responses to one question about synesthesia (from Hartmann, 1991). The question asked “I have regularly experienced an additional (sensory or conceptual) response(s) to a specific stimulus, e.g., colors for letters, words, numbers, sounds, etc.” If participants answered yes, they were prompted for additional information regarding their synesthetic experiences including the location of their concurrent experience.

#### ASMR

The ASMR-15 (Roberts et al., 2019) measures Autonomous Sensory meridian Response (ASMR). The scale has 15 items, with 4 subscales including Sensation (5 items), Altered Consciousness (4 items), Relaxation (3 items), and Affect (3 items). The measure invites participants to indicate their agreement with 15 aspects of the ASMR experience following the statement “When I experience ASMR…” The measure uses a Likert-scale that ranges from 1 (completely untrue for me) to 5 (completely true for me). One open ended question asking about additional triggers follows the 15 items. The measure is scored by summing the responses for the 15 items and taking the mean, after Roberts et al. (2019). Roberts et al. (2019) reported that the measure has a Cronbach’s alpha score of 0.78 and that it also demonstrated good validity.

#### Anomalous experiences

The Survey of Anomalous Experiences (SAE) (Irwin et al., 2013) was employed as a measure of anomalous experiences. This measure results in two scores, one that indicates a tendency to experience anomalies regardless of their interpretation and a second that indicates a tendency to attribute experiences to a paranormal interpretation. Both scores have been found to be associated with good reliability, for experiences Cronbach’s alpha was 0.83, while for paranormal attributions to experiences, the Cronbach’s alpha was 0.78.

#### Psychometry experiences

Psychometry experiences were explored by asking people to respond to one yes/no question about whether they have experienced psychometry. The question was as follows “Some people report that when they handle or are in the presence of objects that were once owned by another person they have accessed information or emotions about that person.” For the qualitative component of this mixed methods design, an open-ended question inquired about the nature of psychometry experiences to those responding yes to the question about psychometry.

### Procedure

An online survey was conducted during November and December of 2021 and lasted for approximately 2 months. A URL that linked to the survey was generated from Qualtrics and distributed firstly among psychology students at UWG (undergraduate, MA and PhD Listservs). Next, the study URL was sent out to faculty and staff in the College of Culture, Art and Scientific Inquiry (CACSI) at the University of West Georgia. The URL was also shared widely on social media and was sent out to members of the Rhine Research Center mailing list. Participants were invited to take part in the study and asked to sign a consent statement followed by a series of questions about anomalous experiences including psychometry. Participants were asked to provide an email address if they wanted to be invited to participate in future related studies. Following the completion of the survey, all participants were thanked for their time.

## Results

### AMSR and anomalous experiences (quantitative analyses)

A reliability analysis was conducted on the ASMR-15 responses in our dataset which found a Cronbach’s alpha score of 0.94, indicating strong internal reliability. Table 1 indicates that psychometry experiencers tended to score higher on ASMR, anomalous experiences and anomalous experiences with a paranormal attribution compared to non experiencers. Those who reported psychometry experiences scored significantly higher on ASMR than those who do not *t*(159) = −3.06, *p* = 0.003. This is equivalent to a Cohen’s d effect size of 0.5.

**Table 1:** Descriptive statistics comparing scores on ASMR and anomalous experiences measures between psychometry experiencers and non-experiencers.

		ASMR (average)	Anomalous experiences (general)	Anomalous experiences with paranormal attribution
Psychometry experiencer	Yes	3.02 (1.07)*N* = 58	12.78 (3.83)*N* = 50	7.78 (5.58)*N* = 50
	No	2.48 (1.05)*N* = 103	9.08 (3.70)*N* = 96	2.73 (3.40)*N* = 96
	Total	2.68 (1.09)*N* = 161	10.35 (4.13)*N* = 146	4.46 (4.89)*N* = 146

In addition, those who report being a psychometry experiencer scored significantly higher on anomalous experiences *t*(159) = −5.86, *p* = 0.001 and anomalous experiences given a paranormal attribution *t*(159) = −6.78, *p* = 0.001. These are equivalent to Cohen’s d effect sizes of 0.98 and 1.2, respectively, (large effects).

A series of Pearson’s correlations were computed to explore how ASMR related to tendencies to report anomalous experiences. ASMR tendencies correlated positively and significantly (two-tailed significance) with proneness to having anomalous experiences (regardless of attribution) *r*(144) = 0.41, *p < 0*.001 and to a slightly lesser extent to anomalous experiences given a paranormal attribution *r*(144) = 0.30, *p* < 0.001. Experience proneness and tendencies to report paranormal experiences correlated positively and significantly with one another, *r*(144) = 0.69, *p* < 0.001.

Table 2 shows that synesthetes tended to score higher than non-synesthetes on ASMR, anomalous experiences and anomalous experiences given a paranormal attribution. Synesthetes were compared with non-synesthetes in terms of scoring on ASMR (total score). Synesthetes scored significantly higher than non-synesthetes on ASMR *t*(161) = −2.98, *p* = 0.003. This reflects a Cohen’s d effect size of 0.5.

**Table 2:** Descriptive statistics comparing synesthetes with non-synesthetes on ASMR and anomalous experiences measures.

		ASMR (average)	Anomalous experiences (general)	Anomalous experiences with paranormal attribution
Synesthesia	Yes	2.96 (1.06)*N* = 74	11.36 (4.26)*N* = 66	5.91 (5.60)*N* = 66
	No	2.43 (1.04)*N* = 89	9.51 (3.84)*N* = 80	3.26 (3.86)*N* = 80
	Total	2.67 (1.08)*N* = 163	10.35 (4.13)*N* = 146	4.46 (4.89)*N* = 146

In terms of anomalous experiences, synesthetes scored higher than non-synesthetes on both anomalous experiences *t*(144) = −2.76, *p* = 0.001 and anomalous experiences given a paranormal attribution *t*(144) = −3.37, *p* = 0.001. The Cohen’s d effect size was 0.46 for experiences and 0.56 for paranormal experiences.

Table 3 demonstrates that in our sample, responding yes to questions about psychometry and synesthesia are less common than those who respond no. The cross tabulation indicates that more people report experiencing neither psychometry or synesthesia than those who report experiencing both types of experience. A chi square calculation was conducted to explore the association. This indicated that these patterns are not statistically significant, *χ*^2^ = 3.10, *df* = 1, *p* = 0.078. Thus, there is no statistical association between frequencies of synesthesia and frequencies of psychometry. This is equivalent to a Cramers V effect size of 0.14.

**Table 3:** Cross tabulation between psychometry experiencers and non-experiencers and synesthesia experiencers and non-experiencers.

		Synesthesia		
		Yes	No	Total
Psychometry	Yes	32	26	58
	No	42	61	103
	Total	74	87	161

### Thematic analysis (qualitative analysis)

Forty-seven people entered a response to the open-ended question pertaining to psychometry experiences. An inductive thematic analysis was undertaken on the responses to the open ended question. This was conducted following the 6 stages articulated by Braun and Clarke (2006). CSM and a student assistant (TS) both engaged with the data and independently identified initial codes and emergent patterns which were later distilled into 5 themes (by CSM) based on the overlaps in meaning units and ensuring that themes were grounded in the data. Illustrative quotes were chosen for each theme. The final list of themes included context; flash of imagery; lived feelings and intense emotions; noesis and perspective taking/empathy. Themes are summarized in Table 4.

**Table 4:** Summary of themes and theme descriptions.

Theme name	Description
Context/schema	Background knowledge and context concerning the objects or physical locations.
Flash of imagery	Vivid (visual and other) imagery that is felt to be coming from a different source to the person and occurring in proximity to the object or location.
Lived feelings and intense emotions	Sudden emotional /energetic experiences experienced in the body (internally) or out in physical space (externally).
Noesis: Direct knowledge and information	Direct knowledge or information that is felt to be received and sometimes later corroborated.
Perspective taking/empathy	Strong connections with another personality and a feeling that the person is experiencing another person’s perspective, via empathy.

Five themes reflect the different aspects of lived experiences associated with psychometry. These themes interact and intersect with each other in several ways.

#### Context

First, the context or schema frames the entire experience, and this occurs either via intention or spontaneously in association with particular locations, spaces or objects. Objects are often known to be associated with a particular deceased individual or objects, physical locations and spaces are understood to be old. As such, there is often an implied historical context that may influence experiences surrounding objects or places. In essence, there is often a definite context or top down influence that contributes to psychometry experiences. Some happen in the context of learning psychometry practice (e.g., as part of a workshop), others happen in the context of knowing that an object belonged to a deceased loved one, and some happen in antiques shops or in thrift stores in which clothing and objects are known to be old and have a story. Other stories share commonality with more classic ideas regarding haunting experiences, and include graveyards and older locations (perhaps known to be haunted or have a difficult history, including the asylum at Milledgeville in Georgia). This theme is present in 34 of the responses shared and is the most prevalent theme. For example, one person noted that “I often purchase objects from thrift stores for their feeling,” another noted that “This happens usually with antiques passed down” another noted that “a remembered incident was touching a gravestone” and another person noted that the experience occurred when “I visited an abandoned mental asylum in Milledgeville” The way that the experience plays out differs from person to person but includes the flash of imagery, intense emotions, noesis and perspective taking/empathy. These experiences do not happen in the same way for each person, but there is a sense that information is acquired that is in association with a particular object or physical location and that the experiences are not coming from the person’s own experiences, imagination or memories.

#### Flash of imagery

When the experience occurs as a flash of imagery, this is felt to be passive and transient, which suggests that the experience is happening in a perceptual like manner that is different from their usual mental imagery. Mental imagery manifests in different sensory modalities, including vivid visual imagery which seems to occur in close temporal proximity to viewing the object or entering a physical space. In addition, the imagery is not felt to be associated with one’s own memory. Visual imagery is passively received and viewed inside the mind’s eye. Sometimes the imagery is experienced as a memory or dream, but it is not felt to be a personal memory. For example, one person noted that “it is usually just a flash of imagery about like seeing segments of a dream” while another noted that images were “pictures flash in my mind’s eye. A bit like a tv,” another noted that “I saw (in my mind’s eye) images of her home in Florida which I had never visited.” Other senses are sometimes evoked in this form of experience, including some sounds or smells, as noted in “sensing a particular smell, even though the object is senseless.” This theme is present in 12 of the responses.

#### Lived feelings and intense emotions

[Sudden] emotions are sometimes experienced in association with given objects. Emotions are intensely experienced and can sometimes co-occur with mental imagery. This can happen in two ways, either in the body (internal experience) or out in the environment, including the feeling of a presence, which aligns with classic experiences of hauntings and after-death communications. For example, for internal experiences one person noted “sometimes it’s just intense feelings and emotions accompanied with a memory or vision of sorts.” Another noted that “it [the object] gave me a sense of joy” while another noted that “I get a bad feeling about the original owner/owners or a good one.” In terms of external experiences, one person noted “I feel like my deceased father was here with me,” indicating that the person could be recognized sometimes. Another noted a less specified experience of “a dark and cold feeling walking around. You always felt like someone was watching you. You could feel the pain and sinister energy.” Another noted that “you could feel emotion left by the house” and finally, in terms of a specific object that “objectively it is a nice vanity. It simply felt…hostile.” This theme is present in 17 of the responses.

#### Noesis: direct knowledge and information

Sometimes, information that is felt to derive from an external source is suddenly or directly received or cognized by the percipient. This is subtly different from the previous themes as the experience pertains to information or knowledge rather than imagery or felt emotions. For example, one person noted that this reflected “a knowing, remembering or confusion,” while another noted that “the information seemed to come from supernatural forces that surround us.” Another noted that this experience was memory like, and noted “I remember moments and can feel them happening or experience a memory of them.” In addition, sometimes the information that is anomalously acquired is sometimes understood to have accuracy or is corroborated, for example, “I later found out that many people had died there.” This theme is present in 15 of the responses.

#### Perspective taking/empathy

The final theme pertains to perspective taking, connection and empathy with the former owner of the object. This might co-occur with some of the other themes, as there may be other attributes to this sense. This theme plays out as descriptions of experiencing another person’s perspective, via empathy. For example, one person noted “I can get overwhelmed and can embody a personality or behavior of an object’s previous owner,” while another noted “it sometimes feels as if I have stepped into their shoes for a minute.” In addition, one person described the experience in a more cognitive manner as “I begin to think about the POV of the object and how it saw the owners lived and spoke to their family.” In terms of the link with empathy, one person directly noted that “I have always been very imaginative and empathetic.” This theme is present in 9 of the responses.

### Integration of quantitative and qualitative findings

The findings from the quantitative part of the study can be complemented by the findings from the TA. ASMR experiences (as measured by the ASMR-15) include a range of triggers, which often include emotional and social information. The presence of intense emotional experiences as one of the emergent themes is in alignment with peoples’ enhanced tendencies toward ASMR. With this in mind, one of the respondents noted that psychometry feels like ASMR when the experience is positive, but different when it is negative. The themes also suggest that experiences that may be more embodied may connect with or influence other experiences at higher cognitive levels. In addition, the existence of empathic experiences also aligns with ASMR. The quantitative and qualitative patterns are further discussed in the discussion section.

## Discussion

Results supported the hypothesis that those who report experiences with psychometry would score significantly higher on the ASMR-15. Our research also found support for the hypothesis that those with a higher level of ASMR would have higher scores on measures of anomalous experiences (with and without paranormal attribution). This is a new finding in the research literature, which should be further explored in future studies.

We also found support for some but not all of our hypotheses concerning synesthesia. There was no association between tendencies to report synesthesia and tendencies to report psychometry. However, synesthetes did score higher than non-synesthetes on anomalous experiences (with and without a paranormal attribution). In addition, and in support of our hypothesis, synesthetes scored significantly higher than non-synesthetes on ASMR. These differences are equivalent to moderate effect sizes.

The association between ASMR and anomalous experiences, including psychometry was expected based on the various correlates of ASMR that suggest that those who are more prone to this type of experiencing may be more likely to exhibit the features of anomaly proneness, including sensitivity and synesthesia-like experiences. These correlations are indicative of moderate effect sizes, suggesting that there is some commonality between these variables, but that there are other factors that may also play a contributing role.

There are several reasons for the association, that we consider may rest on the enhanced sensitivity and empathic tendencies of those who report psychometry experiences. For example, future research might directly include sensory processing sensitivity and empathy as variables of interest for further understanding these experiences. Although empathy was not addressed by the quantitative part of this study, it emerged as a theme in the qualitative component and should be further explored in future studies. This emergent finding aligns with prior research that has found higher empathy scores among those who experience a range of subjective paranormal experiences compared to those who do not (Parra, 2013). Parra (2013) found that those who are more prone to subjective psychic experiences appear to have an enhanced capacity to recognize the emotions of others. This is intriguing in the context of psychometry, given that participants are interacting with objects that were owned by others rather than the individuals *per se*. Empathy is further discussed below, in the context of the results of the thematic analysis.

Likewise, the association between synesthesia and ASMR was expected and is in alignment with prior research that found that synesthesia and ASMR are related to one another (e.g., Poerio et al., 2022b). This is potentially due to the commonalities in the neural architecture and enhanced sensory sensitivity inherent in both variables. After Poerio et al., this association may also implicate ASMR as a form of synsethesia in its own right. A third possibility is that both are unusual experiences which may be connected by a third variable. Further research should unpack the exact nature of the association between these experiences, perhaps from a qualitative perspective to explore how inducers and concurrents deviate and align among those who experience synesthesia and ASMR and those who experience only one type of the two, (given that the association is not perfect).

In the current study, ASMR was more strongly associated with psychometry and anomalous experiences than was synesthesia. However, it should be acknowledged that synesthesia was more crudely assessed here and might have different findings with more systematic assessment. Given the fact that ASMR is more commonly reported than synesthesia (20% compared to 5%), ASMR is a promising variable for further understanding the psychology of anomalous experiences.

In terms of how psychometry experiences relate to other types of anomalous experience, it is interesting to note that psychometry experiencers scored higher on both measures of anomalous experiences, but slightly stronger for experiences that are given a paranormal attribution. This makes sense, given that the concept of psychometry is associated with a paranormal interpretation. Both differences were associated with very strong effect sizes, which is not surprising, given that psychometry is a form of anomalous experience. Future studies might break down the family tree a little more systematically and explore how psychometry relates to the different types of anomalous experiences.

### Limitations

Both synesthesia and psychometry were assessed by asking a question with a dichotomous response choice (yes/no). This may mean that those who responded affirmatively to the synesthesia question may not actually be strong (congenital) synesthetes. Other measures (e.g., the Synesthesia Battery; Eagleman et al., 2007) are designed to test whether one meets test–retest criteria for consistent associations between an inducer and concurrent experience. Future studies might employ more systematic measures of synesthesia to explore how synesthesia and ASMR interact regarding anomalous experiences, including psychometry.

Likewise, psychometry would be better assessed with a more detailed psychometric measure. We consider that this is a first step in exploring these correlates and acknowledge that further research is needed to further explore these experiences and their possible relationship.

Secondly, given that this was an internet-based study, participants self-selected to take part and the sample is not random. In addition, it is possible that having responded to a set of questions about ASMR might have influenced how participants responded to questions about both anomalous experiences and psychometry. In turn, being asked about other forms of anomalous experiences may have served to prime respondents on the psychometry question. However, it is noted that the questionnaire that was employed for this survey asked about different interpretations (including skeptical ones) for neutrally described experiences.

It is still possible that the patterns described herein may reflect those who are more prone to reporting unusual experiences and some of the associations may have been elevated. This is particularly likely given that several respondents received the URL from the mailing list of the Rhine Research Center. As a result, they may have been more likely to report paranormal beliefs and experiences. However, the survey URL was also widely distributed among members of the UWG community who would presumably be less biased toward subjective paranormal experiences and beliefs.

Future research exploring the association between ASMR and different types of anomalous experience is certainly warranted. This might include stand-alone quantitative and qualitative studies as well as other mixed methods designs. The advantages of the current design allow us to garner a richer understanding of psychometry experiences and the reasons for the statistical patterns, due to the convergent nature of this design (quantitative and qualitative data derive from the same sample).

### Qualitative patterns: haunted people syndrome

The qualitative results complement the quantitative findings and indicate that psychometry experiences range in terms of their context and how the experiences manifest. The themes indicate that psychometry experiences occur in a variety of contexts that include intentional and unintentional psychometric practices. As with other forms of anomalous experience, the etiology of anomalous experiences includes a combination of top down and bottom up influences that interact in a systems like manner. This aligns with Haunted People Syndrome (HP-S) (Laythe et al., 2021) which highlights the interactive and constructive nature of anomalous experiences that incorporates transliminality, a range of psychological factors and environmental influences. For example, Laythe et al. (2021) have noted the “interactionist (or enactive) view implies that ghostly episodes involve at least two distinct but related processes: (a) Percipient sensitivity, or “the right people in the right environments (or conditions)” and subsequently, (b) Percipient shaping, or the added influence of an individual’s psychological and social set on the perceptual, attentional, or attributional processes that mediate or dictate the meaning given to anomalies” (Laythe et al., 2021, p. 205). From these data, we cannot ascertain how the physical features of the person-object interactions lead to subjective experiences of psychometry. However, given the range of locations for psychometry experiences, some physical factors are likely to play a role. For example, some people tended to report psychometry experiences in the context of older locations, which aligns more directly with traditional experiences of hauntings. Haunted locations have been associated with several environmental variables (including geomagnetism, lighting effects, etc.) that may be more likely to be detected by sensitive individuals and woven into a ghost narrative (Laythe et al., 2021). Others reported experiences with objects in antiques shops, that may be similar to other haunted locations. Other experiences may include objects that are independent from their wider physical context, but are known to have been owned by a deceased individual. These types of meaningful objects may be more likely to directly influence psychological expectations regarding the object’s former owner via empathy pathways (see later section).

With this in mind, it seems that psychometry experiences can potentially be incorporated into the family tree of ghost related phenomena, whilst also straddling into the domain of extrasensory experiences (in the context of token objects). However, the interactive nature of this experience suggests that it is more enactive than other forms of extrasensory perception, as there is a concrete, physical object with which the person is interacting, rather than more passive ways of experiencing. Although transliminality was not explored within the context of this study, given that ASMR shares several commonalities, including sensitivity and enhanced neural connectivity it is likely that ASMR may play a similar role in the etiology of this form of anomalous experience. The strong imagery associated with these experiences may align with transliminal attributes of these experiencers, given the role of imagery and syncretic cognition in these individuals (Lange et al., 2019). Future research should consider the role of imagery relevant variables in the etiology of psychometry. In addition, future research might explore how transliminality and ASMR interact with regard to psychometry experiences.

### External source

In terms of the phenomenology of psychometry experiences, the thematic analysis indicates that experiences result in a direct perceptual knowing or flash of information that is felt to come from an external source. This sense of externality of source has been noted in the previous literature, in particular in terms of mediumship in which claimants describe another consciousness that they feel is communicating through them (e.g., Roxburgh and Roe, 2013). Roxburgh and Roe note that the source of the information may well be an aspect of the person him or herself, which may reflect hidden aspects of the self. Psychometry has often been studied among special claimants, including mediums. This is an interesting correspondence, given that our study focused on members of the general public, rather than mediums or psychic claimants.

### Different ways of experiencing

Sometimes participants reported an intense emotion was felt in the body or in the physical environment. The differences between these different forms of psychometry experience should be further unpacked in future studies. It is not possible to determine the origins of internal versus external experiences, but this would be an interesting avenue for future research. For example, in the synesthesia literature, projectors experience the concurrent out in physical space, while associators experience the concurrent in the mind’s eye. This seems to be underpinned by different neural processes that underpin these different forms of synesthesia experience (Rouw and Scholte, 2010). Something similar may be occurring among different types of psychometry experiencer. Differences in ways of experiencing may also rest on the context of the experiences, where some are associated with a more general physical substrate (such as a room or building or graveyard) whilst others are focused more on specific objects.

The strong emotional nature of psychometry experiences may also relate to some of the perceptual and sensory biases among those who experience ASMR, given that ASMR is a body based response to interpersonal stimuli. Future research should tease apart the different correlates and etiologies of different ways of experiencing anomalous information in the context of objects.

### Empathy and the role of social cognition

Empathy and perspective taking appear to play a role in psychometry experiences, such that the person experiences the world from the perspective of an unseen other, and subjectively knows that the information is not theirs; i.e., there is a distinct self/other boundary. In empathy research there are assumed to be two components to empathy, a shared representation of the experience of another person by mapping the experience into our own systems and the capacity to distinguish between representations arising in terms of one’s own experience and those arising from the experience of others (Lamm et al., 2016). This imagined self/other distinction in the context of subjective paranormal experiences is interesting and worthy of future study for psychometry and in the context of other psychic experiences. Previous research has found that there is a tendency for “over mentalizing” among those with a proneness toward anomalous experiences. Fyfe et al. (2008) have noted that this may be underpinned by the loose cognitive style that also reflects a tendency to see meaning in randomness (apophenia). This is common among several anomaly prone personality measures including transliminality. In terms of social cognition, Fyfe et al. (2008) argue that this results in seeing more mental states and intentions where none exist. Given that some prior research has found evidence for the acquisition of accurate information in the context of psychometry experiments, future research should further unpack the role of social cognition in psychometry experiences and systematic laboratory studies.

## Conclusion

This study found that ASMR is associated with experiences of psychometry, but there was no statistical relationship between synesthesia and psychometry. However, ASMR and synesthesia were both found to be associated with enhanced tendencies to report anomalous experiences and experiences that are attributed to the paranormal. Qualitative findings suggest that these experiences arise suddenly and in strong imagistic form (across sensory modalities) in the mind’s eye or out in space and seem to be associated with empathy and perspective taking on the part of the experiencer. The findings suggest that the enhanced sensitivity associated with being more prone to ASMR and synesthesia in alignment with some aspects of empathy play a role in the etiology of anomalous experiences in the context of handling objects or places with a history. This can be understood in alignment with Laythe et al.’s (2021) Haunted People Syndrome, which suggests that experiences result via a system of interacting factors including sensitivity, environmental factors and top down influences that result in concrete experiences that are felt to be from a source outside of the person him or herself. We would add that empathy plays a significant additional role in the pathway, and that this may distinguish psychometry experiences from other more traditional forms of haunting phenomena. Future research should further unpack the role of ASMR in a range of anomalous experiences.

## Data availability statement

The raw data supporting the conclusions of this article will be made available by the authors, without undue reservation.

## Ethics statement

This study was approved by the IRB at the University of West Georgia. The studies were conducted in accordance with the local legislation and institutional requirements. The participants provided their written informed consent to participate in this study.

## Author contributions

CS-M: Conceptualization, Formal analysis, Funding acquisition, Investigation, Methodology, Project administration, Writing – original draft, Writing – review & editing.

## Footnotes

^1^Experiments designed to explore whether people can access information that is not available using the normal senses.

^2^Whereby there is a subjective anomalous interaction between a person and the physical environment, without the use of the normal senses.

^3^Those who claim to be able to communicate with the deceased.

^4^Data cleaning consisted of removing incomplete responses. Incomplete responses were defined as those who began the survey but did not respond to any of the individual difference questions, or who began the individual difference questions but stopped after only responding to a few items.

## References

[ref1] Alvarado C. S. (1994). Synesthesia and claims of psychic experiences: an exploratory study [paper presentation]. 37th Annual Convention of the Parapsychological Association, Amsterdam, Netherlands.

[ref9001] Alvaraz B. D. Robertson L. (2013). Synesthesia and binding. in Oxford handbook of synesthesia, Eds. J. Simner and E. Hubbard (Oxford University Press. 317–333.

[ref2] Anderson R. (1984). Psychometry or survival? II. Parapsychol. Rev. 15, 7–10.

[ref3] Baker I. Montague J. Booth A. (2017). A controlled study of psychometry using psychic and non-psychic claimants with actual and false readings using a mixed-methods approach. J. Soc. Psychical Res. 81, 108–122.

[ref5] Barratt E. Davis N. (2015). Autonomous sensory meridian response (ASMR): a flow-like mental state. PeerJ 3:e851. doi: 10.7717/peerj.851, PMID: 25834771 PMC4380153

[ref6] Barratt E. Spence C. Davis N. (2017). Sensory determinants of the autonomous sensory meridian response (ASMR): understanding the triggers. PeerJ. 5:e3846. doi: 10.7717/peerj.3846, PMID: 29018601 PMC5633022

[ref7] Barrington M. R. (2016). ‘Psychometry’. Psi encyclopedia. London: The Society for Psychical Research.

[ref8] Braun V. Clarke V. (2006). Using thematic analysis in psychology. Qual. Res. Psychol. 3, 77–101. doi: 10.1191/1478088706qp063oa

[ref9] Buchanan J. R. (1885). Manual of psychometry: the dawn of a new civilization. 2nd Edn. Boston: Dudley M Holman.

[ref10] Cardeña E. (2020). Derangement of the senses or alternate epistemological pathways? Altered consciousness and enhanced functioning. Psychol. Conscious. Theory Res. Pract. 7, 242–261. doi: 10.1037/cns0000175

[ref11] Cardeña E. Lynn S. J. Krippner S. (2017). The psychology of anomalous experiences: a rediscovery. Psychol. Conscious. Theory Res. Pract. 4, 4–22. doi: 10.1037/cns0000093

[ref12] Cytowic R. E. (1995). Synesthesia: phenomenology and neuropsychology: a review of current knowledge. Psyche 2, Available at: https://philpapers.org/rec/CYTSPA-2

[ref13] Dale L. A. (1944). An informal experiment with Mr. Chester Grady. J. Am. Soc. Psychical Res. 38, 202–221.

[ref14] del Campo M. A. Kehle T. J. (2016). Autonomous sensory meridian response (ASMR) and frisson: mindfully induced sensory phenomena that promote happiness. Int. J. Sch. Educ. Psychol. 4, 99–105. doi: 10.1080/21683603.2016.1130582

[ref15] Denton W. Denton E. M. F. (1871). The soul of things: or, psychometric researches and discoveries. Boston William White and company.

[ref16] Eagleman D. M. Kagan A. D. Nelson S. S. Sagaram D. Sarma A. K. (2007). A standardized test battery for the study of synesthesia. J. Neurosci. Methods 159, 139–145. doi: 10.1016/j.jneumeth.2006.07.012, PMID: 16919755 PMC4118597

[ref9002] Eagleman D. M. (2012). Commentary: Synaesthesia in its protean guises. British Journal of Psychology, 103, 16–19. doi: 10.1111/j.2044-8295.2011.02020.x, PMID: 22229769

[ref17] Engelbregt H. J. Brinkman K. Van Geest C. C. E. Irrmischer M. Deijen J. B. (2022). The effects of autonomous sensory meridian response (ASMR) on mood, attention, heart rate, skin conductance and EEG in healthy young adults. Exp. Brain Res. 240, 1727–1742. doi: 10.1007/s00221-022-06377-9, PMID: 35511270 PMC9142458

[ref18] Fredborg B. K. Champagne-Jorgensen K. Desroches A. S. Smith S. D. (2021). An electroencephalographic examination of the autonomous sensory meridian response (ASMR). Conscious. Cogn. 87:103053. doi: 10.1016/j.concog.2020.103053, PMID: 33232904

[ref19] Fredborg B. Clark J. Smith S. D. (2017). An examination of personality traits associated with autonomous sensory meridian response (ASMR). Front. Psychol. 8, 1–9. doi: 10.3389/fpsyg.2017.00247, PMID: 28280478 PMC5322228

[ref20] Fyfe S. Williams C. Mason O. J. Pickup G. J. (2008). Apophenia, theory of mind and schizotypy: perceiving meaning and intentionality in randomness. Cortex 44, 1316–1325. doi: 10.1016/j.cortex.2007.07.009, PMID: 18635161

[ref21] Hartmann E. (1991). Boundaries in the mind: A new psychology of personality. New York, NY: Basic Books.

[ref22] Hearne K. M. (1989). A questionnaire and personality study of self-styled psychics and mediums. J. Soc. Psychical Res. 55, 404–411.

[ref23] Hettinger J. (1941). Exploring the ultra-perceptive faculty. London: Rider.

[ref24] Hettinger J. (1948). A program for the investigation of psychometry. J. Parapsychol. 12, 90–95, PMID: 18867093

[ref25] Houran J. Lange R. Laythe B. Dagnall N. Drinkwater K. O'Keeffe C. (2019). Quantifying the phenomenology of ghostly episodes: part II – a Rasch model of spontaneous accounts 1. J. Parapsychol. 83, 168–192. doi: 10.30891/jopar.2019.02.05

[ref26] Irwin H. J. (1985). Flight of mind: A psychological study of the out-of-body experience. Metuchen, New Jersey: Scarecrow Press.

[ref27] Irwin H. J. Dagnall N. Drinkwater K. (2013). Parapsychological experience as anomalous experience plus paranormal attribution: a questionnaire based on a new approach to measurement. J. Parapsychol. 77, 39–53.

[ref28] Janik McErlean A. B. Banissy M. J. (2017). Assessing individual variation in personality and empathy traits in self-reported autonomous sensory meridian response. Multisens. Res. 30, 601–613. doi: 10.1163/22134808-00002571, PMID: 31287086

[ref29] Janik McErlean A. B. Banissy M. J. (2018). Increased misophonia in self-reported autonomous sensory meridian response. PeerJ. 6:e5351. doi: 10.7717/peerj.5351, PMID: 30123700 PMC6084287

[ref30] Janik McErlean A. B. Ellis L. Walsh J. (2022). “No pain, no gain”: the impact of autonomous sensory meridian response on pain perception. Perception 51, 565–577. doi: 10.1177/03010066221108273, PMID: 35876369

[ref31] Jawer M. (2006). Environmental sensitivity: inquiry into a possible link with apparitional experience. J. Soc. Psychical Res. 70, 25–47.

[ref32] Kruth J. G. (2015). Five qualitative research approaches and their applications in parapsychology. J. Parapsychol. 79, 219–233.

[ref33] Lamm C. Bukowski H. Silani G. (2016). From shared to distinct self-other representations in empathy: evidence from neurotypical function and socio-cognitive disorders. Philos. Trans. R. Soc. Lond. Ser. B Biol. Sci. 371:20150083. doi: 10.1098/rstb.2015.0083 26644601 PMC4685528

[ref34] Lange R. Houran J. Evans J. Lynn S. J. (2019). A review and reevaluation of the revised transliminality scale. Psychol. Conscious. Theory Res. Pract. 6, 67–89. doi: 10.1037/cns0000153

[ref35] Laythe B. Houran J. Dagnall N. Drinkwater K. (2021). Conceptual and clinical implications of a “haunted people syndrome.”. Spiritual. Clin. Pract. 8, 195–214. doi: 10.1037/scp0000251

[ref36] LeShan L. (1967). A spontaneous psychometry experiment with Mrs. Eileen Garrett. J. Soc. Psychical Res. 44, 14–19.

[ref38] Liester M. B. (2020). Personality changes following heart transplantation: the role of cellular memory. Med. Hypotheses 2020:109468. doi: 10.1016/j.mehy.2019.109468, PMID: 31739081

[ref40] Marwaha S. B. May E. C. (2015). Rethinking extrasensory perception: toward a multiphasic model of precognition. SAGE Open 5:215824401557605. doi: 10.1177/2158244015576056

[ref41] Pagenstecher G. (1920). A notable psychometric test. J. Soc. Psychical Res. 14, 386–417,

[ref42] Parra A. (2013). Cognitive and emotional empathy in relation to five paranormal/anomalous experiences. N. Am. J. Psychol. 15, 405–412.

[ref43] Parra A. Argibay J. C. (2007). Comparing a free-response psychometry test with a free-response visual imagery test for a non-psychic sample. J. Soc. Psychical Res. 71, 91–103

[ref44] Parra A. Argibay J. C. (2008). Reading faces: an experimental exploration of psychometry using photographs and names. Aust. J. Parapsychol. 8:47.

[ref45] Parra A. Argibay J. C. (2009). An experimental study with ordinary people for testing 'Sacred' objects through psi detection. J. Soc. Psychical Res. 73:41.

[ref46] Pearsall P. Schwartz G. E. R. Russek L. G. S. (2002). Changes in heart transplant recipients that parallel the personalities of their donors. J. Near-Death Stud. 20, 191–206. doi: 10.1023/A:1013009425905 10882878

[ref47] Poerio G. L. Blakey E. Hostler T. J. Veltri T. (2018). More than a feeling: autonomous sensory meridian response (ASMR) is characterized by reliable changes in affect and physiology. PLoS One 13:e0196645. doi: 10.1371/journal.pone.0196645, PMID: 29924796 PMC6010208

[ref48] Poerio G. L. Mank S. Hostler T. J. (2022a). The awesome as well as the awful: heightened sensory sensitivity predicts the presence and intensity of autonomous sensory meridian response (ASMR). J. Res. Pers. 97:104183. doi: 10.1016/j.jrp.2021.104183

[ref49] Poerio G. L. Succi A. Swart T. Romei V. &, Gillmeister H. (2023). From touch to tingles: assessing ASMR triggers and their consistency over time with the ASMR trigger checklist (ATC). Conscious. Cogn.;115:103584. doi: 10.1016/j.concog.2023.103584 37820451

[ref50] Poerio G. L. Ueda M. Kondo H. M. (2022b). Similar but different: high prevalence of synesthesia in autonomous sensory meridian response (ASMR). Front. Psychol. 13:990565. doi: 10.3389/fpsyg.2022.990565, PMID: 36248469 PMC9558233

[ref51] Roberts N. Beath A. Boag S. (2019). Autonomous sensory meridian response: scale development and personality correlates. Psychol. Conscious. Theory Res. Pract. 6, 22–39. doi: 10.1037/cns0000168

[ref52] Roberts N. Beath A. Boag S. (2020). A mixed-methods examination of autonomous sensory meridian response: comparison to frisson. Conscious. Cogn. 86:103046. doi: 10.1016/j.concog.2020.103046, PMID: 33242764

[ref53] Roberts N. Beath A. Boag S. (2021). Autonomous sensory meridian response: individual differences and consciousness correlates. Psychol. Conscious. Theory Res. Pract. 8, 27–51. doi: 10.1037/cns0000243

[ref54] Rogo D. S. (1974). Psychometry: getting psychic impressions from objects. Psychic 5, 19–22.

[ref55] Roll W. G. (1966). Further token object tests with a “sensitive.”. J. Am. Soc. Psychical Res. 60, 270–280.

[ref57] Roll W. G. (2003). Review of investigating the paranormal. J. Parapsychol. 67, 187–203.

[ref58] Roll W. G. Joines W. T. (2013). RSPK and consciousness. J. Parapsychol. 77, 192–211.

[ref59] Rouw R. Scholte H. S. (2010). Neural basis of individual differences in synesthetic experiences. J. Neurosci. 30, 6205–6213. doi: w 10.1523/JNEUROSCI.3444-09.2010, PMID: 20445046 PMC6632740

[ref60] Roxburgh E. C. Roe C. A. (2013). “Say from whence you owe this strange intelligence”: investigating explanatory systems of spiritualist mental mediumship using interpretive phenomenological analysis. Int. J. Transpers. Stud. 32, 27–42. doi: 10.24972/ijts.2013.32.1.27

[ref61] Saklani A. (1988). Preliminary tests for psi-ability in shamans of Garhwal Himalaya. J. Soc. Psychical Res. 55, 60–70.

[ref62] Sakurai N. Ohno K. Kasai S. Nagasaka K. Onishi H. Kodama N. (2021). Induction of relaxation by autonomous sensory meridian response. Front. Behav. Neurosci. 15, 1–8. doi: 10.3389/fnbeh.2021.761621, PMID: 34916914 PMC8669134

[ref63] Science of Psychometry: Phenomenal Perceptions (1889). Halls J Health 36, 51–55, PMC9238686 36492149

[ref64] Simmonds-Moore C. (2022). Synesthesia and the perception of unseen realities. J. Humanist. Psychol. 62, 187–207. doi: 10.1177/0022167820918691

[ref65] Simmonds-Moore C. A. Alvarado C. S. Zingrone N. L. (2019). A survey exploring synesthetic experiences: exceptional experiences, schizotypy, and psychological well-being. Psychol. Conscious. Theory Res. Pract. 6, 99–121. doi: 10.1037/cns0000165

[ref66] Simner J. Hubbard E. (2013). Oxford handbook of synesthesia. Oxford: Oxford University Press.

[ref67] Smith S. D. Katherine Fredborg B. Kornelsen J. (2017). An examination of the default mode network in individuals with autonomous sensory meridian response (ASMR). Soc. Neurosci. 12, 361–365. doi: 10.1080/17470919.2016.1188851, PMID: 27196787

[ref68] Smythies J. R. (1987). Psychometry and mescaline. J. Soc. Psychical Res. 54, 266–268.

[ref69] Strang C. B. (2020). Measuring souls: psychometry, female instruments, and subjective science, 1840-1910. Hist. Sci. 58, 76–100. doi: 10.1177/0073275319847065, PMID: 31084223

[ref70] Tart C. T. Smith J. (1968). Two token object studies with Peter Hurkos. J. Am. Soc. Psychical Res. 62, 143–157.

[ref71] Ventola A. Houran J. Laythe B. Storm L. Parra A. Dixon J.. (2019). A transliminal ‘dis-ease’ model of ‘poltergeist agents. J. Soc. Psychical Res. 83, 144–171,

[ref72] Vernon D. (2021). Dark cognition: Evidence for psi and its implications for consciousness. London and New York: Routledge/Taylor & Francis Group.

[ref73] Williams C. (1997). The role of imagination in the construction of anomalous experience [Unpublished doctoral dissertation]. University of Edinburgh.

[ref74] Williams J. M. Blagrove M. (2022). Paranormal experiences, sensory-processing sensitivity, and the priming of pareidolia. PLoS One 17:e0274595. doi: 10.1371/journal.pone.0274595, PMID: 36103566 PMC9473424

